# GRASP55 restricts early-stage autophagy and regulates spatial organization of the early secretory network

**DOI:** 10.1242/bio.058736

**Published:** 2021-10-12

**Authors:** Jennifer Y. Liu, Yu-Hsiu Tony Lin, Andrew M. Leidal, Hector H. Huang, Jordan Ye, Arun P. Wiita, Jayanta Debnath

**Affiliations:** 1 Biomedical Sciences Graduate Program, University of California San Francisco, San Francisco, CA 94143, USA; 2 Department of Laboratory Medicine, University of California San Francisco, San Francisco, CA 94143, USA; 3 Department of Pathology, University of California San Francisco, San Francisco, CA 94143, USA; 4 Department of Laboratory Medicine, University of California San Francisco, San Francisco, CA 94143, USA; 5 Department of Pathology, University of California San Francisco, San Francisco, CA 94143, USA; 6 Department of Laboratory Medicine, University of California San Francisco, San Francisco, CA 94143, USA; 7 Department of Pathology, University of California San Francisco, San Francisco, CA 94143, USA

**Keywords:** Autophagy, GRASP55, Cell biology

## Abstract

There is great interest in understanding the cellular mechanisms controlling autophagy, a tightly regulated catabolic and stress-response pathway. Prior work has uncovered links between autophagy and the Golgi reassembly stacking protein of 55 kDa (GRASP55), but their precise interrelationship remains unclear. Intriguingly, both autophagy and GRASP55 have been functionally and spatially linked to the endoplasmic reticulum (ER)­­-Golgi interface, broaching this compartment as a site where GRASP55 and autophagy may intersect. Here, we uncover that loss of GRASP55 enhances LC3 puncta formation, indicating that GRASP55 restricts autophagosome formation. Additionally, using proximity-dependent biotinylation, we identify a GRASP55 proximal interactome highly associated with the ER-Golgi interface. Both nutrient starvation and loss of GRASP55 are associated with coalescence of early secretory pathway markers. In light of these findings, we propose that GRASP55 regulates spatial organization of the ER-Golgi interface, which suppresses early autophagosome formation.

## Abstract (precis)

**Summary:** The Golgi protein GRASP55 restricts early-stage autophagy and regulates spatial organization of the early secretory network. We also identify a GRASP55 proximal interactome enriched at the ER-Golgi interface.

## INTRODUCTION

Autophagy is an evolutionarily conserved process by which cytoplasmic contents are delivered via double-membraned autophagosomes to lysosomes for degradation (Dikic and Elazar, 2018). While basal autophagy is important for homeostasis (Murrow and Debnath, 2012), autophagy is also potently induced by numerous stress stimuli including nutrient starvation and endoplasmic reticulum (ER) stress (Kroemer et al., 2010). Moreover, dysregulated autophagy is implicated in diverse pathophysiological processes including cancer (Kenific and Debnath, 2015) and neurodegeneration (Nixon, 2013). Hence, further delineating how autophagy is regulated is of great interest to the scientific community. Autophagosome biogenesis follows the progressive steps of initiation, nucleation, expansion, and maturation or lysosomal fusion (Dikic and Elazar, 2018), marking multiple points at which regulation can occur. In recent years, several groups have uncovered links between the Golgi apparatus protein GRASP55 and autophagy (Dupont et al., 2011; Zhang et al., 2018, 2019), although this relationship remains to be fully understood.

The Golgi reassembly stacking protein of 55 kDa (GRASP55) was initially described in mammalian cells as a critical factor for *in vitro* stacking of mitotic Golgi fragments (Shorter et al., 1999) and has canonical roles in Golgi cisternae stacking (Xiang and Wang, 2010), ribbon linking (Feinstein and Linstedt, 2008), and mitotic fragmentation (Duran et al., 2008). However, GRASP55 is not strictly required for bulk flow through the secretory network (Duran et al., 2008; Feinstein and Linstedt, 2008), suggesting additional functions for this protein. Intriguingly, GRASP55 is implicated in unconventional secretion, which also involves the autophagy machinery (Dupont et al., 2011; Gee et al., 2011; Zhang et al., 2015). An ongoing question is whether and how GRASP55 and autophagy are interconnected. The current data is mixed, as GRASP55 has been implicated in regulation of both early (Dupont et al., 2011) and late (Zhang et al., 2018, 2019) stages of autophagy. Further clarification of this question may lie at the ER-Golgi interface.

The ER-Golgi interface includes the interrelated ER exit sites (ERES), from which COPII vesicles containing secretory cargo bud, and the ER-Golgi intermediate compartment (ERGIC), a way station for anterograde and retrograde flow between the ER and Golgi (Brandizzi and Barlowe, 2013). It is also a key regulatory site for autophagy, as COPII proteins are spatially and functionally linked to autophagosome biogenesis (Ishihara et al., 2001; Ge et al., 2013, 2014; Graef et al., 2013; Jeong et al., 2018). Notably, although GRASP55 and its orthologues in other species are classically viewed as Golgi proteins, they are also found at the ER-Golgi interface (Behnia et al., 2007; Levi et al., 2010; Piao et al., 2017). Here, we sought to clarify the relationship between GRASP55 and autophagy and to further ascertain its relationship to the ER-Golgi interface.

## RESULTS AND DISCUSSION

### Loss of GRASP55 enhances early-stage autophagy

**Fig. 1.**
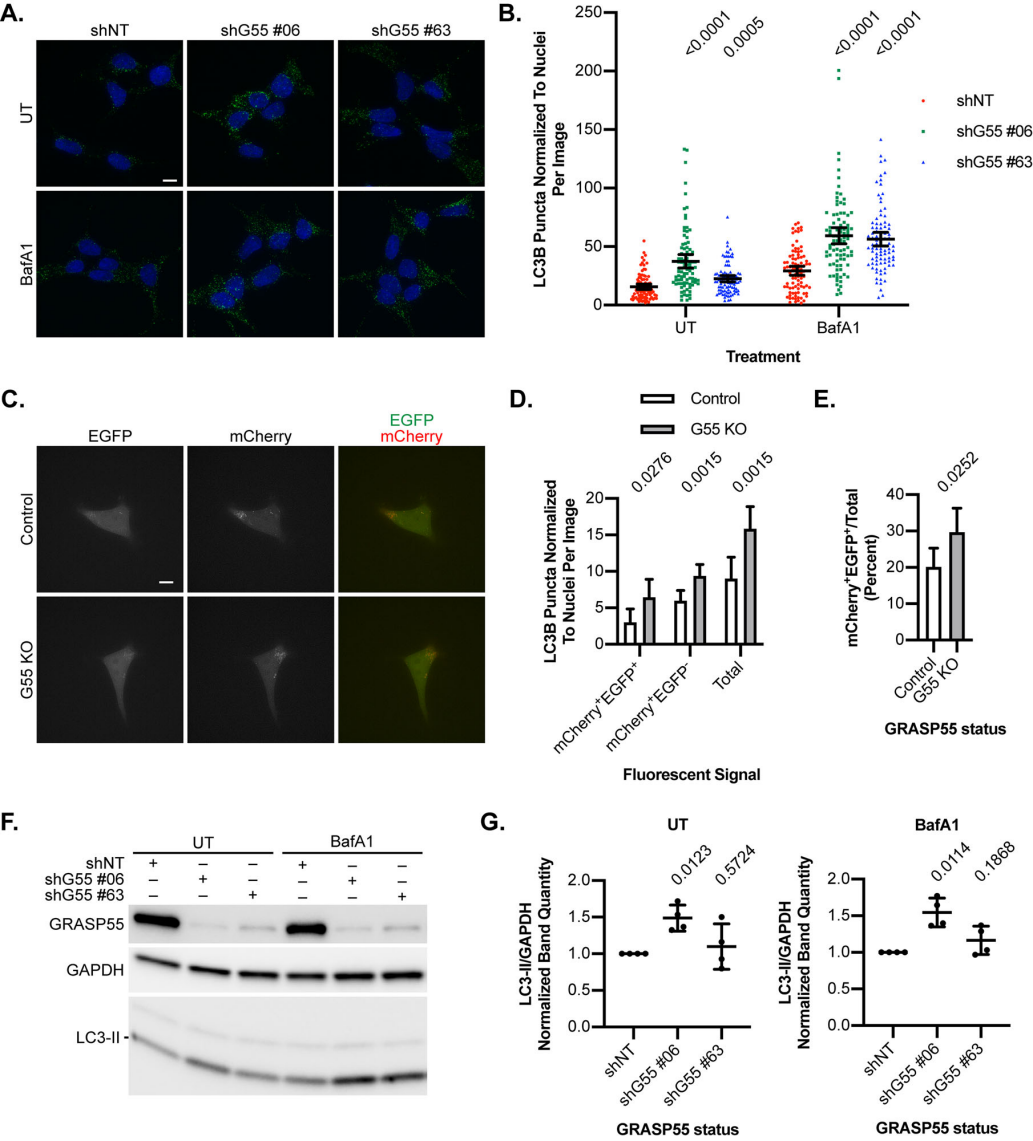
**Effect of GRASP55 depletion on autophagy.** (A) GRASP55 knockdown (shG55 #06 or #63) or non-targeting control (shNT) cells were incubated in EBSS for 60 min with 100 nM bafilomycin A1 (BafA1) or left untreated (UT). Cells were immunostained for LC3B and DAPI-counterstained. Representative images are shown. Scale bar: 10 μm. (B) LC3B puncta were enumerated and normalized to the number of DAPI-stained nuclei on a per-image basis. Welch's ANOVA with Games-Howell's multiple comparisons test was performed within each treatment group, and multiplicity adjusted *P*-values are shown. *n*=90 images per group pooled from three replicates. Error bars: mean±95% confidence interval. (C) GRASP55 knockout (G55 KO) and control cells stably expressing mCherry-EGFP-LC3B were incubated in glucose-free media for 4 h. Live cells were imaged. Representative images are shown. Scale bar: 10 μm. (D) Double-positive (mCherry^+^EGFP^+^), single-positive (mCherry^+^EGFP^−^), and total (mCherry^+^EGFP^+^+mCherry^+^EGFP^−^) LC3B puncta were counted and normalized to the number of nuclei on a per-image basis. A two-tailed unpaired Welch's *t*-test was performed for each group, and *P*-values are shown. *n*=77 images per cell line pooled from three replicates. Error bars: mean±95% confidence interval. (E) The ratio of mCherry^+^EGFP^+^ puncta to total puncta on a per-image basis was calculated and reported as a percentage. A two-tailed unpaired Welch's *t*-test was performed, and the *P*-value is shown. *n*=77 images per cell line pooled from three replicates. Error bars: mean±95% confidence interval. (F) GRASP55 knockdown and control cells were incubated in EBSS for 60 min with 100 nM BafA1 or left untreated. Lysates were analyzed by anti-LC3 immunoblotting. A representative immunoblot is shown. (G) Densitometry of immunoblots from Fig. 1F. LC3-II/GAPDH ratios were calculated, normalizing to shNT controls within each treatment group. One-sample two-tailed *t*-tests were performed with a test value of 1, and *P*-values are shown. *n*=4 bioreplicates. Error bars: mean±s.d.

To investigate the role of GRASP55 in autophagy, we generated stable GRASP55 knockdown and CRISPR-Cas9 deleted HEK293T cells (Fig. S1A,B). Upon culturing cells in amino acid-free media to induce autophagy, the number of LC3B puncta, a marker of autophagosomes, was significantly greater in GRASP55 knockdown cells compared to controls, both in the absence and presence of the lysosomal inhibitor bafilomycin A1 (BafA1) (Fig. 1A,B), suggesting that loss of GRASP55 enhanced the formation of autophagosomes rather than blocked autophagosome-to-autolysosome maturation. These findings support that GRASP55 suppresses autophagy induction.

Intriguingly, our results differed from previous work demonstrating that GRASP55 promoted autophagosome maturation (Zhang et al., 2018, 2019). To further scrutinize the role of GRASP55 in early- versus late-stage autophagy, we used an mCherry-EGFP-LC3B tandem fluorescent reporter to distinguish between double-positive (mCherry^+^EGFP^+^) puncta indicative of autophagosomes and single-positive (mCherry^+^EGFP^−^) puncta indicative of autolysosomes. Previously, the role of GRASP55 in autophagosome maturation was studied in the setting of glucose deprivation (Zhang et al., 2018). We found that, during glucose starvation, loss of GRASP55 was associated with increased mean numbers of both double- and single-positive puncta as well as total puncta (Fig. 1C,D), corroborating that loss of GRASP55 results in increased autophagy induction and overall autophagic flux. Notably, the proportion of double-positive puncta over total puncta was moderately increased in knockout cells (Fig. 1E), suggestive of a relative buildup of autophagosomes due to impaired autophagosome-lysosome fusion. Thus, GRASP55 may have dual roles in both early- and late-stage autophagy, but overall, our results demonstrate that GRASP55 genetic loss results in increased autophagic capacity.

In addition, we asked whether loss of GRASP55 impacted the formation and turnover of LC3-II, the lipidated form of LC3 important for autophagosome biogenesis (Kabeya et al., 2000). GRASP55 knockdown cells were starved in amino acid-free media, with a subset treated with BafA1, and lysates were analyzed by immunoblot (Fig. 1F,G). One of the GRASP55 knockdown lines showed a statistically significant increase in LC3-II relative to control under both untreated and BafA1 conditions; a second line showed a modest effect on LC3-II that was not statistically significant. These findings suggest that loss of GRASP55 modestly enhances LC3-II formation.

We next asked whether the effects of GRASP55 depletion on autophagy arose from increased ER stress. Consistent with previous work (Xiang et al., 2013), we found that diverse ER stress markers were not significantly impacted by GRASP55 knockdown (Fig. S1C,D), suggesting that phenotypic effects of GRASP55 depletion on autophagy are not secondary to ER stress.

### Proximity-dependent biotinylation identifies a GRASP55 proximal interactome enriched at the ER-Golgi interface

**Fig. 2.**
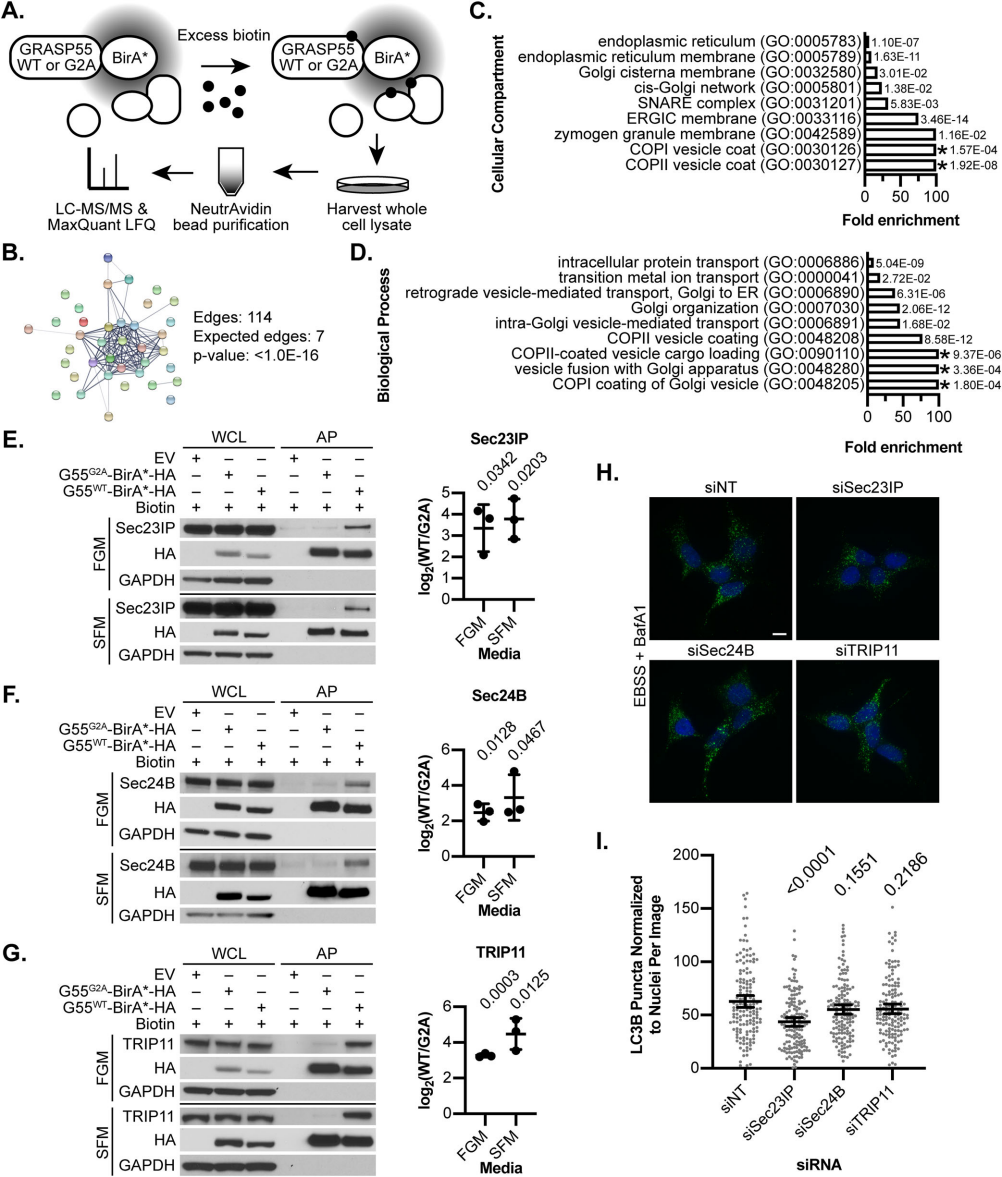
**GRASP55 proximity-dependent biotinylation.** (A) HEK293T cells stably expressing GRASP55^WT^-BirA*-HA or GRASP55^G2A^-BirA*-HA were pulsed with 50 μM biotin to induce proximity-dependent biotinylation. Biotinylated proteins were affinity purified from lysates using NeutrAvidin beads and analyzed by liquid chromatography-tandem mass spectrometry (LC-MS/MS) followed by by label-free quantification (LFQ) using MaxQuant software. (B) Hits from the GRASP55 proximal interactome were analyzed for functional protein-protein interactions (PPI) using the STRING database tool. Putative functional relationships are indicated by network edges connecting protein nodes, with strength of support represented by line thickness. The PPI enrichment *P*-value reported by STRING is shown. (C,D) Gene ontology enrichment analysis of cellular compartment and biological process terms. A Fisher's exact test with false discovery rate (FDR) correction was performed to evaluate statistical significance. Fold enrichment of the most specific subclass terms using the hierarchy sort function with FDR <0.05 are shown, along with individual FDR values. *: greater than 100-fold enrichment. ER: endoplasmic reticulum; ERGIC: endoplasmic reticulum-Golgi intermediate compartment. (E,F,G) Immunoblots for selected proteins from the GRASP55 proximal interactome were performed. HEK293T cells stably expressing wild-type (G55^WT^-BirA*-HA, WT) or mutant (G55^G2A^-BirA*-HA, G2A) GRASP55 BioID constructs or empty vector control (EV) were incubated with 50 μM biotin for 24 h in full growth media (FGM) or serum-free media (SFM). Whole cell lysates (WCL) were harvested and affinity purified (AP) for biotinylated proteins, and immunoblot analysis was performed as indicated. Representative immunoblots are shown. Band densitometry was performed on AP samples, and the WT to G2A band intensity ratios were log_2_-transformed. One-sample two-tailed *t*-tests were performed with a test value of 0, and *P*-values are shown. *n*=3 bioreplicates. Error bars: mean±s.d. H. GRASP55 knockdown HEK293T cells (shG55 #06) were transiently depleted of selected proteins from the GRASP55 proximal interactome using siRNAs against Sec23IP, Sec24B, and TRIP11 or a non-targeting (NT) control. Double-knockdown cells were incubated in EBSS with 100 nM bafilomycin A1 (BafA1) for 60 min and stained for LC3B along with DAPI. Representative images are shown. Scale bar: 10 μm. I. LC3B puncta were counted and normalized to the number of DAPI-stained nuclei on a per-image basis. Welch's ANOVA with Games-Howell's multiple comparisons test was performed, and multiplicity adjusted *P*-values are shown. *n*=150 images per group pooled from three replicates. Error bars: mean±95% confidence interval.

To gain insight into the activities of GRASP55 mediating its intracellular roles, we used proximity-dependent biotinylation (BioID) (Roux et al., 2012; Roux et al., 2013) to elucidate the physical neighbors of GRASP55 and further delineate its localization and potential interacting partners. Constructs encoding GRASP55 fused to an HA-tagged promiscuous biotin ligase BirA* (Roux et al., 2012) were stably introduced into HEK293T cells, using the G2A mutant form of GRASP55, which is defective in Golgi localization (Shorter et al., 1999), as a control (Fig. 2A; Fig. S2A). Immunoblot and immunofluorescence analysis confirmed that these constructs had unique biotinylation profiles and distinct intracellular localization patterns (Fig. S2B,C,D), supporting their utility in identifying proximal neighbors of GRASP55. Lysates from cells expressing BioID constructs under biotin-supplemented serum-free media conditions were affinity purified for biotinylated proteins and subject to liquid chromatography-tandem mass spectrometry followed by label-free quantification analysis to identify protein interactors. From this dataset, we identified a subset of hits enriched at least twofold in GRASP55^WT^-BirA*-HA-expressing cells over GRASP55^G2A^-BirA*-HA-expressing cells with a *P*-value≤0.05, thus representing a putative GRASP55 proximal interactome (Table S1, Fig. S2E). Importantly, this interactome included several previously identified GRASP55 physical interactors, as annotated on the protein-protein interaction database BioGRID (Oughtred et al., 2019) (Fig. S2F).

To ascertain functional interrelationships among proteins in the GRASP55 proximal interactome, we performed STRING analysis (Szklarczyk et al., 2019), which identified statistically significant protein-protein interaction enrichment within our dataset (Fig. 2B). Furthermore, gene ontology analysis (Ashburner et al., 2000; Mi et al., 2019; Gene Ontology Consortium, 2021) using cellular compartment (Fig. 2C) and biological process (Fig. 2D) terms revealed several highly enriched terms associated with the ER-Golgi interface, suggesting that GRASP55 has activities in this cellular sub-compartment. We verified a subset of hits using immunoblot analysis and found that Sec23IP, Sec24B, and TRIP11 were reliably biotinylated by GRASP55^WT^-BirA*-HA-expressing cells (Fig. 2E,F,G). While these proteins have previously characterized functions in the early secretory pathway (Tani et al., 1999; Jensen and Schekman, 2011; Roboti et al., 2015), we asked whether they also played a role in GRASP55-dependent regulation of autophagy. GRASP55 knockdown cells were co-depleted for Sec23IP, Sec24B, or TRIP11 (Fig. S2H) and subject to amino acid starvation in the presence of BafA1. Depletion of Sec23IP, but not of Sec24B or TRIP11, reversed the increase in LC3B puncta found in GRASP55 knockdown cells (Fig. 2H,I). Rescue of Sec23IP expression (Fig. S2I) increased the number of LC3B puncta (Fig. S2J), corroborating these findings are unlikely due to off-target effects.

Sec23IP contains a proline-rich N-terminal region important for its interaction with Sec23, while the central region and C-terminus of Sec23IP exhibit high sequence homology with phospholipase A1 (Tani et al., 1999). Further investigation revealed that Sec23IP localizes to ERES and is recruited to membranes in a Sar1-dependent manner, similar to Sec23 (Shimoi et al., 2005). Depletion of Sec23IP caused dispersal of COPII markers, suggesting that Sec23IP is required for organization of ERES (Shimoi et al., 2005), and delayed export from the ER using a vesicular stomatitis virus glycoprotein transport assay, indicating that Sec23IP plays a functional role in the early secretory pathway (Ong et al., 2010). Specifically, Sec23IP also interacts with Sec31A, suggesting a model in which Sec23IP tethers the inner and outer subcomplexes of the COPII coat (Ong et al., 2010). Moreover, Sec23IP was found to promote ERES formation by coupling recognition of phosphatidylinositol 4-phosphate to dissociation of Sec16A from the COPII machinery (Klinkenberg et al., 2014). On a physiological level, Sec23IP has been implicated in diverse processes including spermiogenesis (Arimitsu et al., 2011), steroidogenesis (Aghazadeh et al., 2020), neurodevelopmental disorders (Reuter et al., 2017), adult attention deficit hyperactivity disorder (Zayats et al., 2016), neural crest defects (McGary et al., 2010), melanoma (Rose et al., 2011), and bone strength (Alam et al., 2009). Our results here support that Sec23IP interacts proximally with GRASP55 and functionally contributes to the ability of GRASP55 to suppress early-stage autophagy.

Our BioID screen also revealed the presence of autophagy-related proteins SQSTM1, ATG9A, ATG2B, ATG3, and WIPI2, which did not meet the criteria for inclusion in the GRASP55 proximal interactome (Table S2). Intriguingly, these proteins were not preferentially biotinylated by one BioID construct over the other, suggesting that the interactions of GRASP55 with autophagy-related proteins may be independent of its localization to the Golgi membrane. Notably, GRASP55 was previously shown to interact with Beclin-1 (Zhang et al., 2019). While Beclin-1 was not detected by mass spectrometry in our screen, immunoblot analysis of BioID lysates revealed that Beclin-1 was modestly preferentially biotinylated by GRASP55^WT^-BirA*-HA-expressing cells over controls (Fig. S2G). These findings support that GRASP5 localizes proximally to elements of the autophagy machinery.

### Starvation and loss of GRASP55 result in coalescence of the ER-Golgi interface

**Fig. 3.**
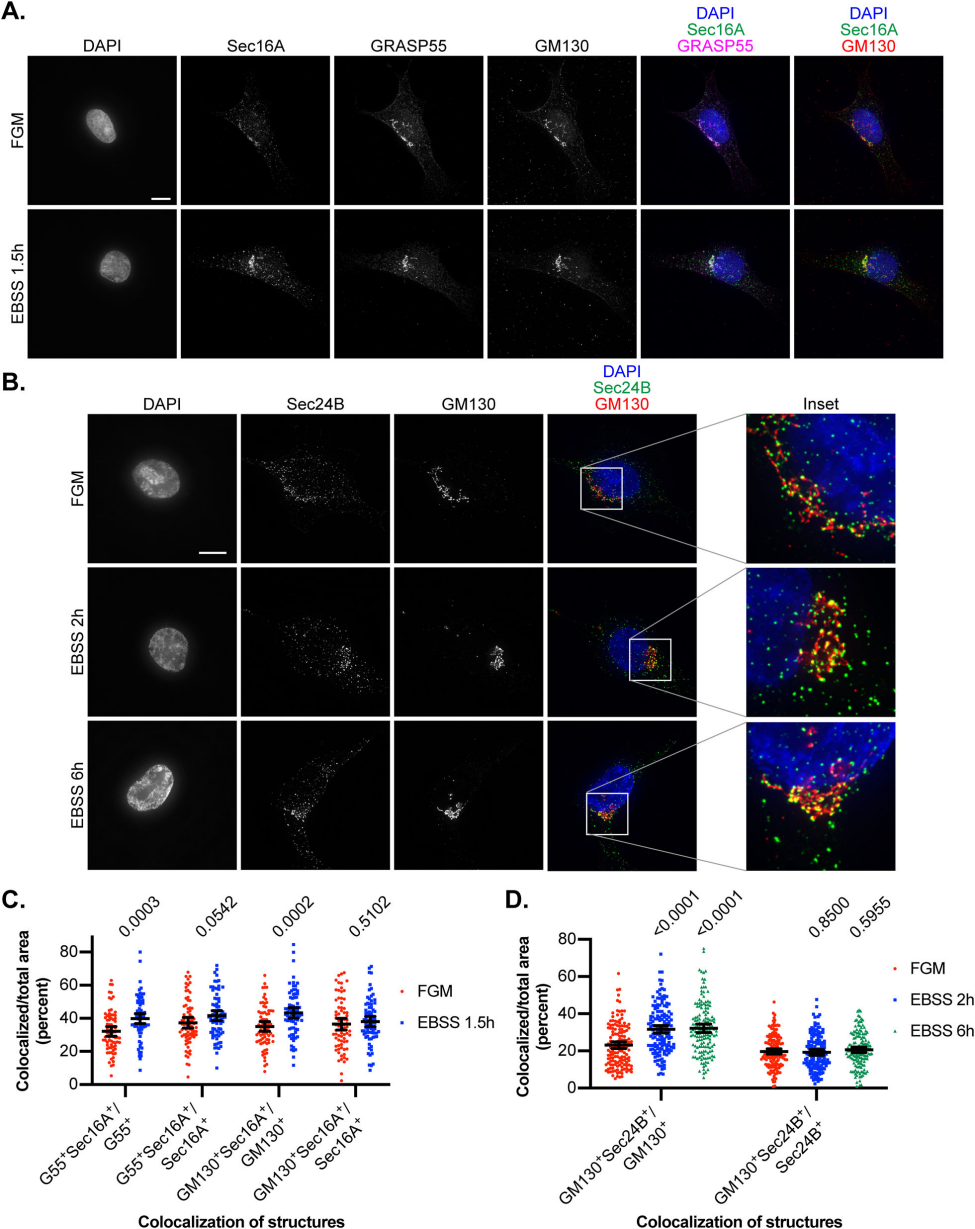
**Effect of starvation on the ER-Golgi interface.** (A) HEK293T cells were incubated in EBSS for 1.5 h or maintained in full growth media (FGM) and stained for GRASP55, GM130, and Sec16A along with DAPI. Representative images are shown. Scale bar: 10 μm. (B) HEK293T cells were incubated in EBSS for indicated times or maintained in FGM and stained for GM130 and Sec24B along with DAPI. Representative images are shown. Scale bar: 10 μm. (C) Analysis of immunofluorescence results from Fig. 3A. Areas covered by GRASP55 (G55), GM130, and Sec16A as well as the overlapping regions between GRASP55 or GM130 with Sec16A were measured using ImageJ. The ratio of overlapping region to individual area of a structure was calculated as indicated and reported as a percent. Unpaired two-tailed *t*-tests were performed, and *P*-values are shown. *n*=74 FGM cells and 76 EBSS cells, pooled from two experiments. Error bars: mean±95% confidence interval. (D) Analysis of immunofluorescence results from Fig. 3B. The areas covered by GM130 and Sec24B, including overlapping regions, were measured using ImageJ. The ratio of overlapping region to individual area of a structure was calculated and reported as a percent. One-way ANOVA with Dunnett's multiple comparisons test was performed for each colocalization group, and multiplicity adjusted *P*-values are shown. *n*=149 FGM cells, 150 EBSS 2 h cells, and 150 EBSS 6 h cells, pooled from three replicates. Error bars: mean±95% confidence interval.

Previous work collectively indicates that COPII trafficking from ERES to the ERGIC is both induced by starvation and required for autophagy (Ge et al., 2013, 2014; Jeong et al., 2018). Because the GRASP55 proximal interactome is highly enriched at the ER-Golgi interface, we sought to better understand the relationship between GRASP55 and the ER-Golgi interface during starvation. Cells were amino acid-starved and analyzed by immunofluorescence microscopy for the ERES marker Sec16A, *cis-*Golgi marker GM130, and GRASP55 (Fig. 3A). Starvation was associated with a statistically significant increase in the percentage of either GRASP55 or GM130 overlapped by Sec16A; in contrast, converse ratios of the percentage of Sec16A overlapped by GRASP55 or GM130 were modestly elevated by starvation, and these differences were not statistically significant (Fig. 3C). Similar results were observed for the COPII marker Sec24B and GM130 at additional longer starvation timepoints (Fig. 3B,D). Together, these findings indicate that starvation induces a spatial reorganization of the early secretory pathway characterized by increased coalescence of the ER-Golgi interface.

**Fig. 4.**
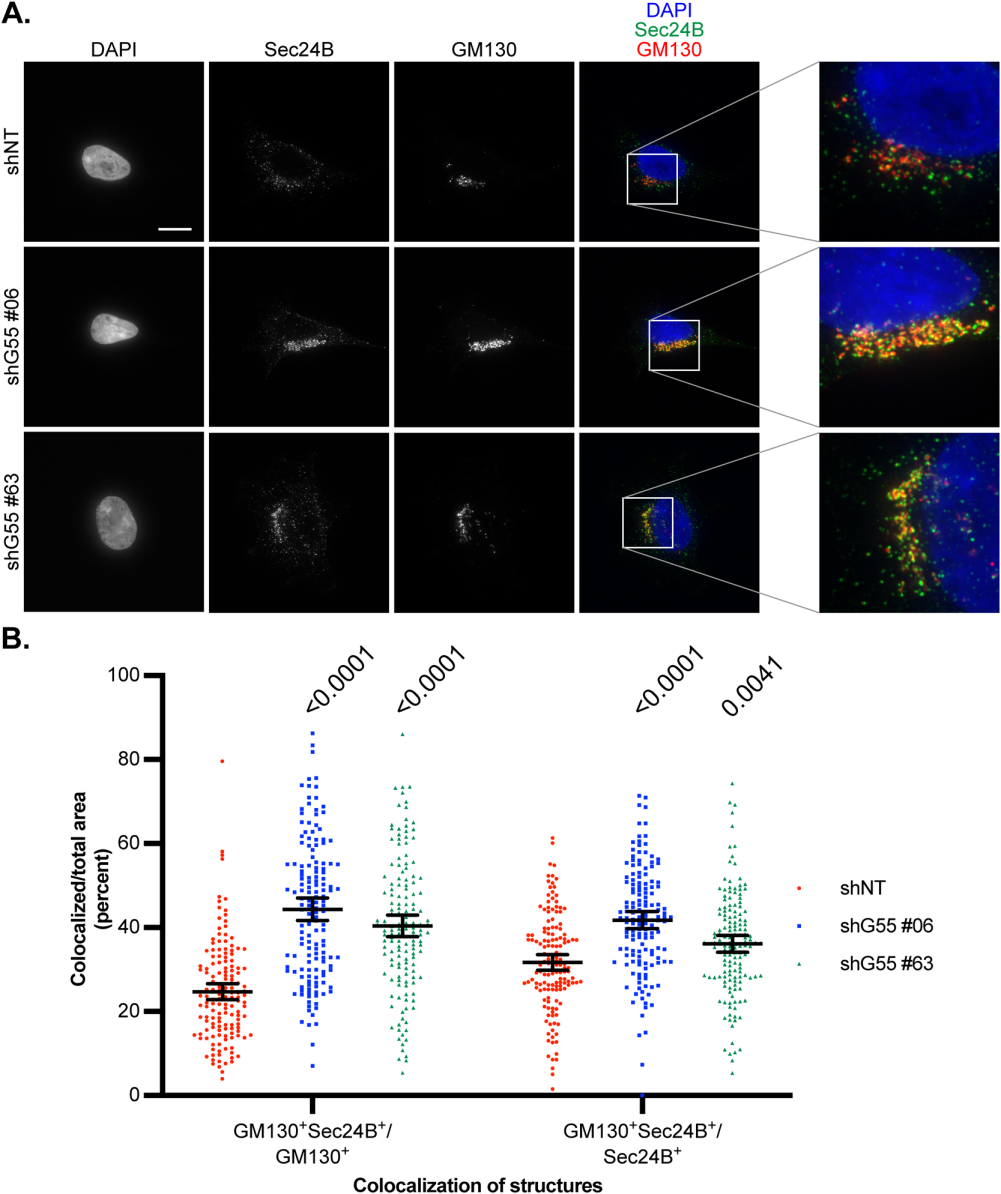
**Effect of GRASP55 depletion on the ER-Golgi interface.** (A) GRASP55 stable knockdown (shG55 #06 and #63) and non-targeting control (shNT) cells were stained for GM130 and Sec24B along with DAPI. Representative images are shown. Scale bar: 10 μm. (B) The areas covered by GM130 and Sec24B, including overlapping regions, were measured using ImageJ software. The ratio of overlapping region to individual area of a structure was calculated and reported as a percent. Welch's ANOVA with Games-Howell's multiple comparisons test was performed for each colocalization group, and multiplicity adjusted *P*-values are shown. *n*=150 cells pooled from three replicates. Error bars: mean±95% confidence interval.

We next asked whether loss of GRASP55 affected spatial organization of the early secretory network. Others have demonstrated that loss of GRASP55 increases membrane association of the COPII protein Sec31, suggesting that GRASP55 negatively regulates trafficking at ERES (Xiang et al., 2013). Using immunofluorescence microscopy, GRASP55 knockdown cells were stained for Sec24B and GM130 and compared to controls (Fig. 4A). Remarkably, loss of GRASP55 was associated with a reliable increase in both overlap of GM130 by Sec24B and overlap of Sec24B by GM130 (Fig. 4B). Similar results were observed for Sec16A and GM130 (Fig. S3A,B). Thus, similar to amino acid starvation, loss of GRASP55 enhances coalescence of early secretory network markers.

In conclusion, our work supports a role for GRASP55 in negatively regulating early autophagosome formation, in addition to its previously described function in promoting autophagosome-to-autolysosome maturation (Zhang et al., 2018, 2019). It is possible that these dual roles serve to temporally coordinate different stages of autophagy; further work is required to clarify the precise stages in early autophagy impacted by GRASP55. We also demonstrate that GRASP55 is both spatially and functionally associated with the ER-Golgi interface, where it limits the coalescence of early secretory pathway markers. Because trafficking at the ER-Golgi interface is required for efficient autophagy (Ge et al., 2013, 2014; Jeong et al., 2018) and GRASP55 negatively regulates trafficking (Xiang et al., 2013), we postulate that GRASP55 restricts early autophagy by limiting trafficking at the ER-Golgi interface. Finally, we have identified a GRASP55 proximal interactome enriched at the ER-Golgi interface which includes the COPII-associated protein Sec23IP. Our studies suggest that GRASP55-dependent restriction of early-stage autophagy also involves Sec23IP; future work is necessary to determine the precise mechanism underlying this interaction.

## MATERIALS AND METHODS

### Cell culture

HEK293T cells (ATCC CRL-3216) and derivative lines were cultured in full growth media (FGM) comprised of Dulbecco's Modified Eagle Medium (DMEM) with high glucose and sodium pyruvate (Thermo Fisher Scientific, 11995065) supplemented with 10% heat-inactivated fetal bovine serum (FBS) (Atlas Biologicals, F- 0500-D), 25 mM HEPES Buffer (Teknova, H1030), and 100 U ml^−1^ penicillin and 100 μg ml^−1^ streptomycin (Thermo Fisher Scientific, 15140122). Cells were incubated at 37°C and 5% CO_2._ HEK293T cells were authenticated and tested for mycoplasma contamination (IDEXX). Starvation was performed by washing cells 1-2 times and replacing with starvation media. Serum-free media (SFM) was prepared by omitting FBS. Amino acid starvation media was prepared by supplementing Earle's Balanced Salt Solution (EBSS) (GE Healthcare Life Sciences, SH30029.02) with 25 mM HEPES. Glucose-free media was prepared by supplementing glucose- and sodium pyruvate-free DMEM (Thermo Fisher Scientific, 11966025) with 2% FBS, 25 mM HEPES, and 100 U ml^−1^ penicillin and 100 μg ml^−1^ streptomycin. When indicated, the following compounds were supplemented as follows: bafilomycin A1 (Sigma-Aldrich, B1793, 100 nM), brefeldin A (Sigma-Aldrich, B6542, 10 μg ml^−1^), thapsigargin (Sigma-Aldrich, T9033, 1 μM), tunicamycin (Sigma-Aldrich, T7765, 5 μg ml^−1^).

### Genetic constructs

Human GRASP55 (Gorasp2) was cloned from mRNA isolated from HEK293T cells that was reverse transcribed using AccuScript High Fidelity Reverse Transcriptase (Agilent) and amplified using PfuUltra II Hot Start DNA Polymerase (Agilent) with GRASP55-specific primers (forward: agctggatccgccatgggctcctcgcaaagcg; reverse: agctgaattcttaaggtgactcagaagcattg). pcDNA3.1 MCS-BirA(R118G)-HA was a gift from Kyle Roux (Addgene Plasmid #36047; http://n2t.net/addgene:36047; RRID:Addgene_36047). To generate GRASP55^WT^-BirA*-HA, GRASP55 cDNA was amplified using Phusion High Fidelity DNA Polymerase (Thermo Fisher Scientific) and GRASP55-specific primers to remove the GRASP55 stop codon (forward: agctaccggtgccgccatgggctcctcgcaaagcgtcgaga; reverse: agctggatccaggtgactcagaagcattggc) and subcloned into pcDNA3.1 MCS-BirA(R118G)-HA between AgeI and BamHI restriction sites in-frame with BirA(R118G)-HA. To generate GRASP55^G2A^-BirA*-HA, site-directed mutagenesis using the QuikChange II XL kit (Agilent 200521) was performed with GRASP55^WT^-BirA*-HA as a template and primers designed to induce a Glycine (G)-to-Alanine (A) mutation at amino acid residue 2 (forward: gtgccgccatggcctcctcgcaaag; reverse: ctttgcgaggaggccatggcggcac). To generate retroviral expression vectors, the GRASP55^WT^-BirA*-HA and GRASP55^G2A^-BirA*-HA sequences were first amplified using the Phusion High-Fidelity PCR kit (Thermo Fisher Scientific) with flanking primers (forward: cgcaaatgggcggtaggcgtg; reverse: agctctcgagctatgcgtaatccggtacatc). PCR products were then cut at AgeI and XhoI restriction sites flanking the constructs, while pBABE-puro GFP-LC3 (Addgene Plasmid #22405) was cut at AgeI and SalI restriction sites flanking the GFP-LC3 sequence. Subsequently, GRASP55^WT^-BirA*-HA and GRASP55^G2A^-BirA*-HA were subcloned into pBABE between AgeI and SalI sites, fully replacing GFP-LC3. Constructs were verified by sequencing. The pBABE-puro mCherry-EGFP-LC3B plasmid previously constructed in the Debnath lab (Addgene plasmid # 22418; http://n2t.net/addgene:22418; RRID:Addgene_22418). Human Sec23IP cDNA construct (SC126781) and PCMV6-XL5 empty vector control (PCMV6XL5) were obtained from OriGene. pLKO.1 lentiviral expression vectors containing GRASP55 short hairpin RNA (shRNA) inserts were obtained from Sigma-Aldrich as follows: non-targeting control (shNT) (Sigma-Aldrich, SHC002); shGRASP55 #06 (shG55 #06) (Sigma-Aldrich, TRCN0000278406, sequence: ccgggacctcagtcacaccaagtaactcgagttacttggtgtgactgaggtctttttg); and shGRASP55 #63 (shG55 #63) (Sigma-Aldrich, TRCN0000278363, sequence: ccgggatctgctgaaagcaaacgttctcgagaacgtttgctttcagcagatctttttg).

### CRISPR-Cas9 gene editing

sgRNA sequences targeting GRASP55 (tggccgccccacaggttact) or a scramble sequence (gcactaccagagctaactca) were cloned into pSpCas9(BB)-2A-Puro (PX459), a gift from Feng Zhang (Addgene plasmid # 48139; http://n2t.net/addgene:48139; RRID:Addgene_48139) (Ran et al., 2013). Plasmids were transfected into HEK293Ts, and then 24-48 h after transfection, cells were selected with 1 μg ml^−1^ puromycin for 48 h. Genomic DNA from polyclonal populations was prepared using QuickExtract (Epicentre) and assessed by Surveyor analysis (IDT 706020). Cells were seeded into single-cell populations in 96-well plates, and clones were identified and expanded. Knockout of GRASP55 protein was verified by immunoblot.

### Viral packaging and infection

shRNA vectors were packaged into virus using a third-generation lentiviral packaging system. Briefly, packaging plasmids and shRNA expression vectors were transfected into HEK293T cells using Lipofectamine LTX and Plus Reagent (Thermo Fisher Scientific, 15338100) and incubated overnight. The next day, transfection media was removed and replaced with fresh media. The following day, media containing virus was removed and clarified using a 0.45 µm filter. Retrovirus was generated by transfecting pBABE expression vectors into Phoenix-AMPHO retroviral packaging cells (ATCC CRL-3213) using polyethylenimine (PEI), collecting virus-containing media 2 days afterwards, and then clearing through a 0.45 µm filter. To infect cells, viral media was diluted in FGM containing 8 µg ml^−1^ polybrene and added to sparsely seeded cells; the next day, virus-containing media was removed and cells were allowed to recover for an additional day before selection with 1 µg ml^−1^ puromycin for 2–3 days or until uninfected control cells were fully killed.

### Transient gene silencing

siRNA pools directed against Sec23IP (Horizon Discovery L-012370-01-0005), Sec24B (Horizon Discovery L-008299-02-0005), TRIP11 (Horizon Discovery L-012684-00-0005) or non-targeting control (Horizon Discovery D-001810-10-05) were introduced into cells with Dharmafect 1 transfection reagent (Horizon Discovery T-2001-02). For co-transfections, individual siRNA directed against Sec23IP 3′UTR (Horizon Discovery J-012370-10-0005) or non-targeting control (Horizon Discovery D-001810-01-05) were introduced along with specified plasmids using Lipofectamine 2000 transfection reagent (Thermo Fisher Scientific, 11668019). Cells were analyzed 3 days after transfection.

### Antibodies and conjugated reagents

Commercially available antibodies were used as follows; citations and validation information may be found on the manufacturer's website where available. For immunoblotting, the following reagents were used: rabbit anti-HA tag [Cell Signaling Technology (CST) 3724, 1:1000, lot 8], mouse anti-GAPDH (EMD Millipore MAB374, 1:10,000 or 1:20,000, lot 3109750), rabbit anti-GORASP2 (Proteintech, 10598-1-AP, 1:2000, lot 00022940), rabbit anti-LC3B (CST 3868, 1:1000, lot 14), rabbit anti-TRIP11 (Proteintech, 26456-1-AP, 1:1000, lot 00043643), rabbit anti-Sec24B (CST 12042, 1:1000, lot 1), rabbit anti-Sec23IP (Atlas Antibodies, HPA043305, 1:1000, lot R39611), rabbit anti-Beclin-1 (CST 3738S, 1:1000, lot 3), mouse anti-HA tag (CST 2367, 1:1000, lot 5), Streptavidin-HRP (Thermo Fisher Scientific, 21130, 1:20,000, lot SB241752A), rabbit anti-α-tubulin (CST 2125, 1:5000, lot 11), mouse anti-ATF6 (Abcam, ab122897, 1:500, lot GR111809-71), rabbit anti-IRE1α (CST 3294, 1:1000, lot 11), rabbit anti-eIF2α (CST 9722, 1:1000, lot 15), rabbit anti-phospho-IRE1 (Abcam, ab124945, 1:2500, lot GR271918-24), rabbit anti-phospho-eIF2α (CST 9721, 1:1000, Lot 21), Peroxidase AffiniPure Donkey Anti-Rabbit IgG (Jackson ImmunoResearch, 711-035-152, 1:5000), Peroxidase AffiniPure Donkey Anti-Mouse IgG (Jackson ImmunoResearch, 715-035-150, 1:5000). For immunofluorescence and fluorescence microscopy, the following reagents were used: rabbit anti-LC3B (CST 3868, 1:200, lot 14), rabbit anti-GORASP2 (Proteintech, 10598-1-AP, 1:250, lot 00022940), mouse anti-GM130 (BD Biosciences, 610823, 1:50, lot 8033959), rabbit anti-HA tag (CST 3724, 1:100, lot 8), mouse anti-GRASP55 (Abcam, ab211532, 1:250, lot GR273456-2), sheep anti-GM130 (Novus Biologicals, AF8199, 10 µg ml^−1^, lot CIEQ0117051), rabbit anti-Sec16A (Atlas, Antibodies, HPA005684, 1:100, lot A84463), rabbit anti-Sec24B (CST 12042, 1:100, lot 1), DyLight Streptavidin-549 (Vector Laboratories, SA-5549, 1:250, lot ZC0830), Alexa Fluor 488 donkey anti-mouse (Invitrogen, A-21202, 1:1000, lot 2018296), Alexa Fluor 488 donkey anti-rabbit (Invitrogen, A-21206, 1:1000, lot 1981155), Alexa Fluor 568 donkey anti-mouse (Invitrogen, A10037, 1:1000, lot 2026157), Alexa Fluor 647 donkey anti-mouse (Invitrogen, A-31571, 1:1000, lot 2045337), Alexa Fluor 647 donkey anti-rabbit (Invitrogen, A-31573, 1:1000), Alexa Fluor 647 donkey anti-sheep (Invitrogen, A-21448, 1:1000, lot 2045339).

### Lysate preparation

To prepare whole cell lysates, cells were washed with Dulbecco's phosphate-buffered saline (DPBS) (Thermo Fisher Scientific, 14190144) over ice, harvested in lysis buffer dependent on the application, and spun at 14,000 rpm in a microcentrifuge to remove the pellet. Generally, lysis buffer was prepared by supplementing RIPA buffer (Sigma-Aldrich, R0278) with 10 mM sodium fluoride, 10 mM β-glycerophosphate, 1X protease inhibitor cocktail (PIC) (Sigma-Aldrich, P8340), 10 mM sodium orthovanadate, 0.5 mM PMSF (Sigma-Aldrich, P7626), 10 nM Calyculin A (Sigma-Aldrich, C5552), 10 µg ml^−1^ Pepstatin A (Sigma-Aldrich, P4265), and 10 µg ml^−1^ E64D (Calbiochem, 330005). For BioID immunoblots, lysis buffer was prepared by supplementing RIPA buffer with 1 mM EGTA, 1 mM EDTA, 1 mM β-glycerophosphate, 10 mM sodium fluoride, 2.5 mM sodium pyrophosphate, 1 mM sodium orthovanadate, and 1X PIC. Protein concentration was quantified by BCA assay (Thermo Fisher Scientific, 23223 and 23224).

### Immunoblotting

Samples were prepared for sodium dodecyl sulfate-polyacrylamide gel electrophoresis (SDS-PAGE) by adding protein sample buffer supplemented with β-mercaptoethanol to lysates and heating for 5 min at 95°C (or as described elsewhere). Protein samples were resolved by SDS-PAGE and transferred to polyvinylidene difluoride (PVDF) membranes (Bio-Rad, 1620177). Following transfer, membranes were briefly checked for transfer using Ponceau stain (Sigma-Aldrich, P7170) when necessary. Membranes were blocked for approximately 0.5–1 h with 5% milk (LabScientific, M-0841) or bovine serum albumin (BSA) (Sigma-Aldrich, A3059) in phosphate-buffered saline with 0.1% Tween-20 (PBST), incubated with primary antibody diluted in 5% milk or BSA either overnight at 4°C or 1 h at room temperature, washed extensively with PBST, incubated with secondary antibody diluted in 5% milk or BSA either overnight at 4°C or 1 h at room temperature, and then washed again with PBST. Alternatively, when using Streptavidin-HRP, membranes were blocked with 5% BSA in PBST, incubated with Streptavidin-HRP in 1% BSA, washed extensively with PBST, incubated for 5 min with blocking buffer (PBST supplemented with 10% FBS and 1% Tween-20), and washed again with PBST, modified from previously established protocols (Roux et al., 2013). Membranes were visualized by incubating with enhanced chemiluminescence (ECL) reagent (Thermo Fisher Scientific, 32106, Bio-Rad 1705060, or EMD Millipore WBLUF0100) and developed on film or on a ChemiDoc Imaging System (Bio-Rad). When necessary, membranes were stripped using harsh stripping buffer with β-mercaptoethanol and re-probed. Band densitometry analysis was performed using ImageJ software (Version 1.52) or Bio-Rad Image Lab software (version 6.0.1).

### Immunofluorescence and fluorescence microscopy

Cells were seeded at 20,000 cells per well onto glass coverslips coated with 10 µg ml^−1^ fibronectin and allowed to attach overnight. Following any experimental treatments or incubations, cells were fixed with 4% paraformaldehyde for 15 min, quenched with 300 mM glycine for 5 min and permeabilized with 0.1% Triton X-100 (or ice-cold 100% methanol for LC3B puncta experiments) for 10 min, all at room temperature. DPBS was used as a diluent for immunofluorescence reagents. To assess BioID construct localization, cells were blocked in 0.2% BSA for 30 min, incubated with primary antibody diluted in 0.2% BSA for 1 h, washed with DPBS, incubated with secondary antibody diluted in 0.2% BSA for 1 h protected from light, and washed again. To assess biotin localization, cells were blocked in 1% BSA for 30 min, incubated with DyLight Streptavidin-549 diluted in 1% BSA for 1 h protected from light, and washed with DPBS. To assess intracellular protein localization, cells were blocked in 3% donkey serum for 1 h, incubated with primary antibody diluted in 3% donkey serum for 1 h, washed with DPBS, incubated with secondary antibody diluted in 3% donkey serum for 1 h protected from light, and washed again. Coverslips were mounted to slides using ProLong Gold Antifade Mountant with DAPI (Thermo Fisher Scientific, P36935) and allowed to cure overnight. Slides were imaged with a DeltaVision microscope system (Applied Precision) outfitted with a 60X NA 1.42 objective (Olympus) and a 100X NA 1.40 objective (Olympus). Images were acquired, deconvolved, and cropped with softWoRx (Applied Precision) and processed and analyzed in ImageJ dependent on the application.

### Immunofluorescence image processing and analysis

For LC3B puncta analysis, a Z-stack of images for each channel was taken, images were deconvolved and cropped, and a max intensity projection was created. ImageJ macros were developed to rapidly analyze individual images for each experiment. In brief, for each experiment, images for the LC3B channel were adjusted for brightness and contrast using a consistent scale, background was removed, images were manually thresholded using consistent parameters or auto-thresholded using the MaxEntropy algorithm, and particles were counted. For LC3B puncta number analysis, the number of LC3B particles was normalized to the number of whole nuclei manually counted on a per-image basis. For Golgi and ERES colocalization analysis, a Z-stack of images for each channel was taken, images were deconvolved and cropped, and a max intensity projection was created. ImageJ macros were developed to rapidly analyze individual images for each experiment. In brief, the extracellular space was excluded dependent on the experiment, then images for each channel other than DAPI were auto-thresholded using the MaxEntropy algorithm, and the size of particles within the cell was measured to determine the area covered by each structure. To determine the area of overlap between two structures, a region of interest was created based on the localization pattern of one structure, and then the portion of the second structure contained within the region of interest was measured. The percent colocalized area between two structures A and B was calculated by dividing the area of colocalization (A^+^B^+^) by the total area of each structure (A^+^ or B^+^) in turn, e.g. A^+^B^+^/A^+^ and A^+^B^+^/B^+^.

### mCherry-EGFP-LC3B tandem reporter imaging

GRASP55 CRISPR knockout cells and control cells stably expressing mCherry-EGFP-LC3B were seeded at 80,000 cells per dish onto fibronectin-coated 35 mm glass bottom dishes (MatTek P35G-1.5-14-C) and allowed to attach overnight. The next day, cells were washed and incubated in glucose-free media for 4 h. Live cells were imaged on a DeltaVision microscope system at 37°C. Images were deconvolved and cropped as described above and then processed in ImageJ using an automated macro. Briefly, for each channel, background was removed, images were auto-thresholded using the RenyiEntropy algorithm, and the number of particles within a specified size parameter was counted. The Colocalization plugin (Bourdoncle, 2004) was then run to identify areas of overlap between masks of the two images, and the number of colocalized points (mCherry^+^EGFP^+^) was counted. The number of mCherry^+^EGFP^−^ puncta was determined by subtracting the number of mCherry^+^EGFP^+^ puncta from the total number of mCherry^+^ particles, and these numbers were normalized to the number of nuclei with detectable puncta on a per-image basis. Images that contained fewer than two puncta normalized to the number of nuclei were excluded from data analysis.

### Proximity-dependent biotinylation and affinity purification

Cells expressing BioID constructs were induced to undergo proximity-dependent biotinylation and processed for downstream applications based on established protocols (Roux et al., 2012, 2013). For immunoblot analysis, HEK293T cells stably expressing BioID constructs or empty vector were seeded to equivalent confluency on tissue culture dishes and then incubated for 24 h in media with or without 50 µM biotin (Sigma-Aldrich, B4501). Cell lysates were harvested in RIPA lysis buffer and quantified by BCA assay. For each intended downstream immunoblot, 0.5 mg lysate was mixed with 25 µl triply pre-washed NeutrAvidin bead slurry (Thermo Fisher Scientific, 29204) on a tube rotisserie overnight, then washed extensively with RIPA lysis buffer to remove unbound protein, mixed with 37.5 µl 3X protein sample buffer, and heated at 95°C for 30 min to elute captured protein before SDS-PAGE and immunoblotting. To induce biotinylation for fluorescence microscopy, HEK293T cells stably expressing BioID constructs were seeded onto coverslips as described above and incubated for 24 h in media with 50 µM biotin before processing for imaging. To induce biotinylation for mass spectrometry analysis, HEK293T cells stably expressing BioID constructs were each seeded to equivalent confluency on four 15 cm tissue culture dishes and the next day washed, refed with SFM supplemented with 50 µM biotin, and incubated for 24 h. Samples were then prepared based on a previously established protocol (Couzens, 2013) with modifications, as follows. Cells from each construct were scraped into ice-cold PBS and pooled, centrifuged at 4°C for 5 min at 230×***g***, washed with ice-cold PBS, and lysed in 2 ml modified RIPA lysis buffer (RIPA buffer supplemented with 1 mM EGTA, 1 mM EDTA, and 1X PIC). Lysates were then sonicated over ice and centrifuged twice at 14,000 rpm for 30 min at 4°C in a microcentrifuge to remove insoluble material. Protein concentration was determined and equalized by adding additional modified RIPA lysis buffer to the more concentrated sample. Equivalent amounts of lysate were incubated with 100 µl triply pre-washed NeutrAvidin bead slurry and mixed on a tube rotisserie at 4°C overnight. The next day, the beads were washed once with modified RIPA lysis buffer, twice with 50 mM HEPES-KOH pH 8.0 supplemented with 100 mM KCl, 10% glycerol, 2 mM EDTA, and 0.1% NP-40, and three times with 50 mM ammonium bicarbonate pH 8.0 before further preparation for mass spectrometry.

### Mass spectrometry sample preparation

Half of the beads and affinity purified proteins were resuspended in 50 mM ammonium bicarbonate buffer pH 8.0. Disulfide bonds were reduced with 10 mM tris(2-carboxyethyl)phosphine (Sigma-Aldrich, C4706), and free thiols were alkylated with 40 mM 2-chloroacetamide (Sigma-Aldrich, 22790) at room temperature for 30 min. Proteins on beads were quantified by 280 nm absorbance on a Nanodrop (Thermo Fisher Scientific). Subsequently, approximately 250 μg of bound proteins were trypsinized on-bead with 2.5 μg of mass spectrometry grade trypsin (Thermo Fisher Scientific, 90057) dissolved in 50 mM acetic acid, and incubated at room temperature overnight, for 20–24 h, on a rotisserie. After digestion, trypsinized peptides were harvested by centrifuging beads at low speed (3000×***g***) for 10 min. The supernatant was transferred to a clean Lo-bind centrifuge tube (Eppendorf), the beads were then washed with an additional 100–200 μl 0.1% Trifluoroacetic acid (TFA), centrifuged again, and the supernatant was combined with the initial fraction. Peptides were acidified to a final concentration of 0.5% trifluoroacetic acid (pH<3) and desalted by SOLA C18 solid phase extraction (SPE) cartridge (Thermo Fisher Scientific, 60109-001), and then dried down in a speed-vac. Dried peptides were stored at −20°C, and resuspended in 2% acetonitrile, 0.1% formic acid in a bath sonicator for 5 min to a concentration of 0.2 μg μl^−1^ before MS analysis.

### Liquid chromatography-tandem mass spectrometry (LC-MS/MS) analysis

For each sample, a total of 1 µg of peptides was injected into a Dionex Ultimate 3000 NanoRSLC instrument with a 15-cm Acclaim PEPMAP C18 (Thermo Fisher Scientific, 164534) reverse phase column coupled to a Thermo Q-Exactive Plus Mass Spectrometer. The samples were separated on a 3.5-h non-linear gradient using a mixture of Buffer A (0.1% FA) and B (80% ACN/0.1% FA). The initial flow rate was 0.5 µl min^−1^ at 3% B for 15 min followed by a drop in flow rate to 0.2 µl min^−1^ and a non-linear increase (curve 7) to 40% B for the next 195 min. The flow rate was then increased to 0.5 µl min^−1^ while Buffer B was linearly ramped up to 99% for the next 6 min. Finally, we maintained the peak flow rate and Buffer B concentration for another 7 min before dropping the concentration back to 3%. The MS survey scan was performed over a mass range of 350–1500 m/z with a resolution of 70,000. The automatic gain control (AGC) was set to 3e6, and the maximum injection time (MIT) was 100 ms. We performed a data-dependent MS2 acquisition at a resolution of 17,500, AGC of 5e4, and MIT of 150 ms. The 15 most intense precursor ions were fragmented in the HCD at a normalized collision energy of 27. Dynamic exclusion was set to 20 s to avoid over-sampling of highly abundant species.

### Mass spectrometry data processing

Raw spectral data was analyzed using MaxQuant (version 1.5.1.2) to identify and quantify proteins. Default software settings were used, and data was searched against ∼80,000 human proteins from Uniprot (https://www.uniprot.org). The ‘proteinGroups’ output file was processed for downstream analyses in R (version 3.4.0). First, proteins annotated as ‘reverse,’ ‘only identified by site,’ or ‘potential contaminant’ were filtered out. To remove poorly quantified proteins, quantification was required in at least two replicates in one condition. In addition, proteins that had a single observation in one condition that was greater than the mean of two or more observations in the other condition were omitted. The intensity data derived from label-free quantification by MaxQuant was then log_2_-transformed. Missing values were imputed using a hybrid imputation approach, ‘MLE’ setting for values missing at random and ‘MinProb’ for those missing not at random (*imputeLCMD* package). Finally, Welch's two-tailed unpaired *t*-test was performed to evaluate the statistical significance of the change in biotinylated proteins between WT and G2A groups.

### Proximal interactome analysis

GRASP55 BioID protein hits with label-free quantification intensity≥twofold enriched in WT over G2A samples and *P*-value of ≤0.05 by Welch's two-tailed unpaired *t*-test were further processed to remove ambiguous or obsolete terms as follows: first, for protein groups with multiple possible identities, the first identity was chosen; ambiguous identities were clarified; and protein groups that contained only identities which were listed as obsolete in the UniProt protein database (https://www.uniprot.org) (The UniProt Consortium, 2018) at the time of writing were removed. The GRASP55 proximal interactome list was compared to previously identified interactors of GRASP55 using BioGRID (version 3.5.184) (https://thebiogrid.org) (Oughtred et al., 2019). Data was also assessed for functional protein interactions using the STRING database (version 11.0) (https://string-db.org) (Szklarczyk et al., 2019). For this analysis, the minimum required interaction score was set to the default setting of a medium confidence score (0.400). Additionally, data was analyzed for gene ontology enrichment using the Gene Ontology Consortium GO Enrichment Analysis tool (geneontology.org) against the *Homo sapiens* reference list (PANTHER Overrepresentation Test release date 20200407, GO Ontology database release date 2020-02-21) (Ashburner et al., 2000; Mi et al., 2019; Gene Ontology Consortium, 2021).

### Statistical analysis

Unless noted otherwise, statistical analyses were performed using Prism software (Version 8) (GraphPad). Sample size was defined for each experiment based on the effect size and variance for the experimental approach. Statistical methods were performed as described in figure legends.

## Supplementary Material

Supplementary information
