# Supplementary material for: GRASP55 restricts early-stage autophagy and regulates spatial organization of the early secretory network
Source: Biol Open. 2021 Oct 12;10(10):bio058736. doi: 10.1242/bio.058736 (PMC8524720; doi:10.1242/bio.058736)
Supplement: Supplementary information [file biolopen-10-058736-s1.pdf]

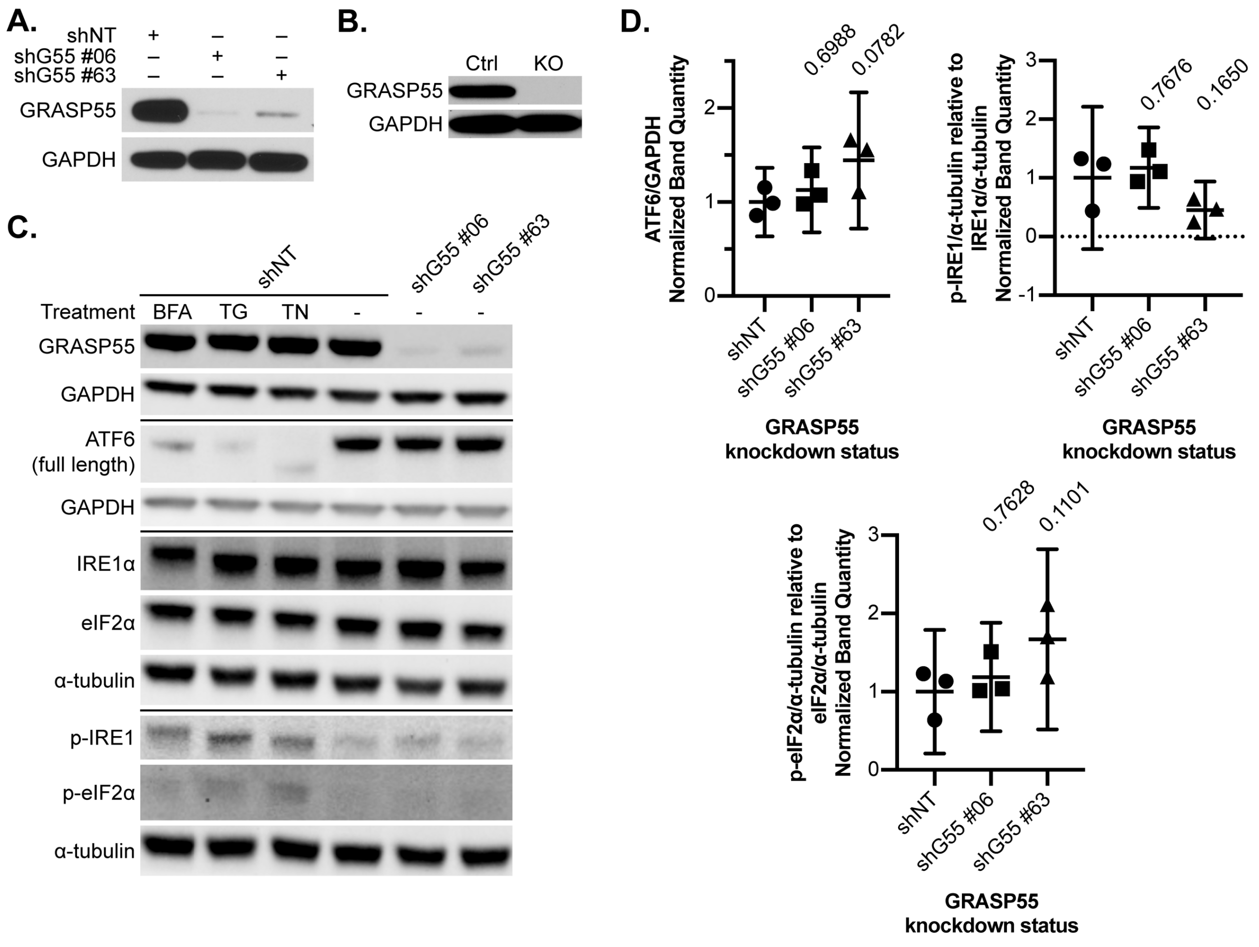

Fig. S1. Effect of GRASP55 depletion on autophagy

A. Lentiviral shRNA expression vectors targeting GRASP55 or a non-targeting control (shNT) were introduced into HEK293T cells and selected for stable incorporation with puromycin. Lysates were analyzed by immunoblot, and efficient knockdown was achieved by two separate hairpins (shG55 #06 and shG55 #63).

B. GRASP55 CRISPR-Cas9 knockout cells (KO) were generated using a GRASP55-targeting sgRNA. Control cells (Ctrl) were generated using a scramble sgRNA.

C. Lysates from HEK293Ts stably expressing GRASP55 shRNA or shNT control were analyzed by immunoblot for expression of ER stress markers. As a positive control for ER stress, shNT cells were treated for 5 hours with 10 μg ml^{-1} brefeldin A (BFA), 1 μM thapsigargin (TG), or 5 μg ml^{-1} tunicamycin (TN). A representative immunoblot is shown. D. Band densitometry was performed on ER stress immunoblots, and band ratios were calculated as indicated, normalizing to mean control values. One-way ANOVA with Dunnett's multiple comparisons test was performed, and multiplicity adjusted p-values are shown. n = 3 bioreplicates. Error bars: mean ± 95% confidence interval.

Biology Open • Supplementary information

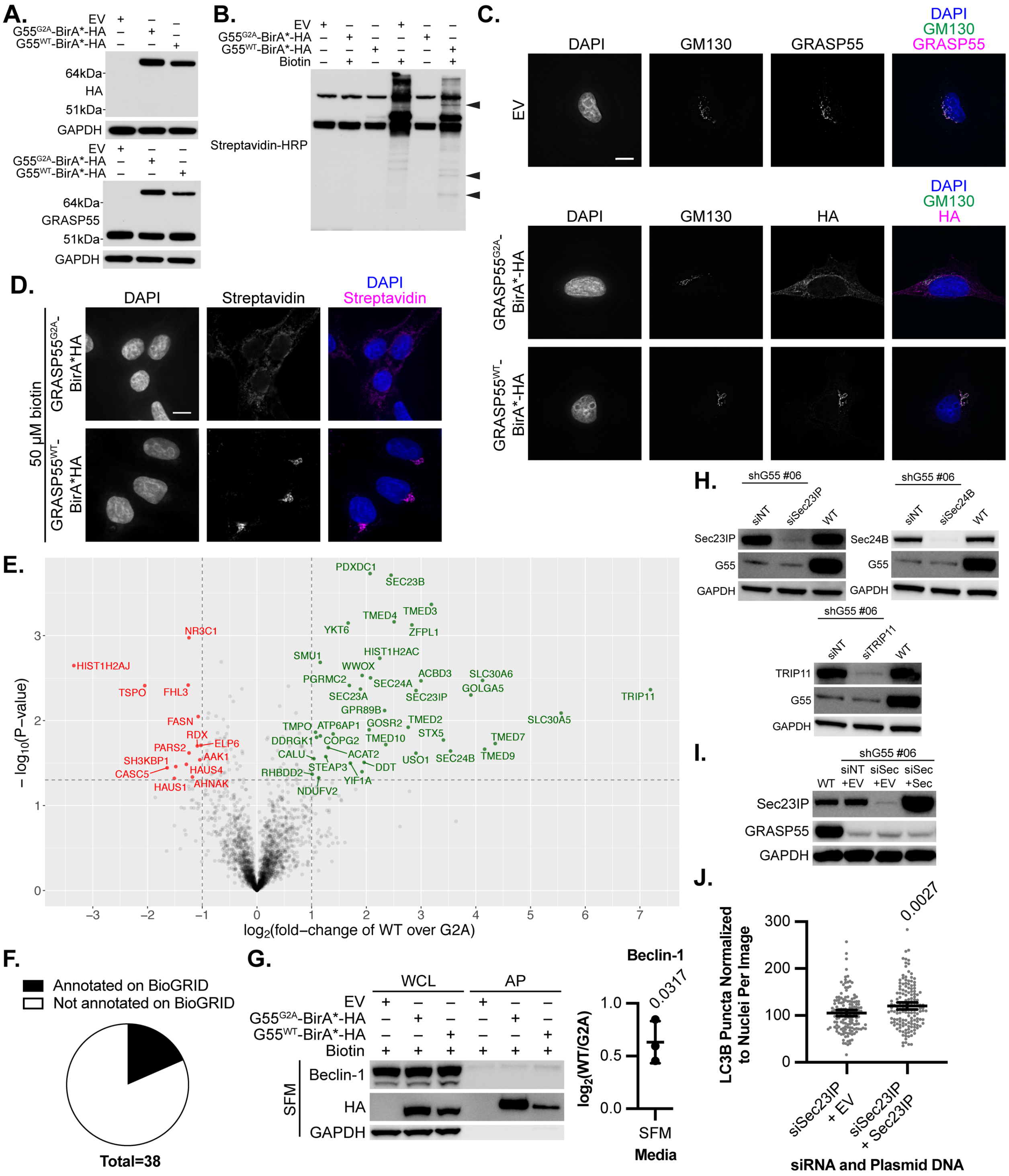

Biology Open • Supplementary information

Fig. S2. GRASP55 proximity-dependent biotinylation

A. HEK293Ts were retrovirally transduced to stably express GRASP55 (G55)^{WT}-BirA*-HA, GRASP55 (G55)^{G2A}-BirA*-HA, or empty vector control (EV). Lysates were harvested from cells incubated with biotin, and expression of constructs was confirmed by immunoblot.

B. HEK293Ts stably expressing GRASP55^{WT}-BirA*-HA , GRASP55^{G2A}-BirA*-HA, or empty vector BioID constructs were incubated in media with (+) or without (–) 50 μM biotin for 24 hours. Biotinylated proteins were affinity purified from lysates and analyzed by western blot using Streptavidin-HRP to detect biotinylated proteins. Arrows indicate bands distinct to the GRASP55^{WT}-BirA*-HA construct. A representative blot is shown.

C. HEK293Ts stably expressing GRASP55 BioID constructs or EV control were co-stained for the Golgi marker GM130, GRASP55, and/or HA tag (as indicated) along with DAPI. Representative images are shown. Scale bar = 10 μm.

D. HEK293T cells stably expressing GRASP55 BioID constructs were incubated in media with 50 μM biotin for 24 hours and co-stained with DAPI and fluorophore-conjugated Streptavidin. Representative images are shown. Scale bar = 10 μm.

E. Volcano plot displaying all protein hits identified by GRASP55 proximity-dependent biotinylation. Label-free quantification intensity ratios in GRASP55^{WT}-BirA*-HA (WT) over GRASP55^{G2A}- BirA*-HA (G2A) samples were $\log_2$-transformed, and a Welch's two-tailed unpaired t-test was performed to assess statistical significance. Hits with ≥ 2-fold enrichment in WT over G2A and p-value ≤ 0.05 are shown in green, while hits with ≥ 2-fold enrichment in G2A over WT and p-value ≤ 0.05 are shown in red. n = 3 bioreplicates.

Biology Open • Supplementary information

F. Top hits from the GRASP55 proximal interactome were compared to the list of GRASP55-interacting proteins annotated in the BioGRID protein database.

G. HEK293T cells stably expressing $G55^{WT}$-BirA*-HA (WT) or $G55^{G2A}$-BirA*-HA (G2A) GRASP55 BioID constructs or EV control were incubated with 50 μM biotin for 24 hours in serum-free media (SFM). Whole cell lysates (WCL) were harvested and affinity purified (AP) for biotinylated proteins, and immunoblot analysis was performed as indicated. Representative immunoblots are shown. Band densitometry was performed on AP samples, and the WT to G2A band intensity ratios were log_2-transformed. A one-sample two-tailed t-test was performed with a test value of 0, and p-value is shown. n = 3 bioreplicates. Error bars: mean ± standard deviation.

H. siRNAs targeting Sec23IP, Sec24B, and TRIP11 were used to generate double-knockdown cells in HEK293Ts stably expressing shRNA against GRASP55 (shG55 #06). Immunoblots were performed to confirm knockdown alongside non-targeting (siNT) and wild-type (WT) controls. Representative immunoblots are shown.

I. siRNA targeting Sec23IP 3'UTR (siSec) and plasmid DNA containing Sec23IP cDNA (Sec) were co-transfected into GRASP55 stable knockdown cells (shG55 #06). Immunoblots were performed to confirm expression using WT, siNT, and EV controls. Representative immunoblots are shown.

J. GRASP55 knockdown HEK293T cells (shG55 #06) were transiently depleted of Sec23IP using individual siRNA targeted against the 3'UTR and co-transfected with EV or Sec23IP cDNA. Cells were incubated in EBSS with 100 nM bafilomycin A1 for 60 minutes and stained for LC3B along with DAPI. LC3B puncta were counted and normalized to the number of DAPI-stained nuclei on a per-image basis. Welch's two-tailed unpaired t-test was performed, and p-value is shown. n = 150 images per group pooled from 3 replicates. Error bars: mean ± 95% confidence interval.

Biology Open • Supplementary information

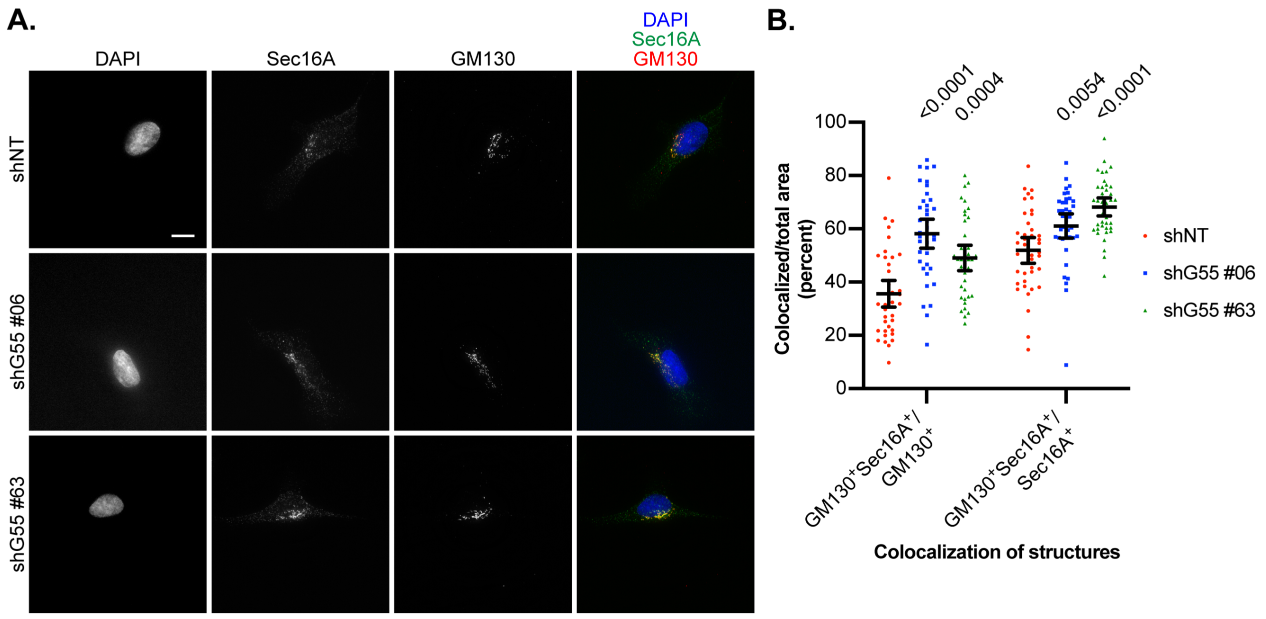

Fig. S3. Effect of GRASP55 depletion on the ER-Golgi interface

A. GRASP55 stable knockdown (shG55 #06 and #63) and non-targeting control (shNT) cells were stained for GM130 and Sec16A along with DAPI. Representative images are shown. Scale bar = 10 µm.

B. The areas covered by GM130 and Sec16A, including overlapping regions, were measured using ImageJ software. The ratio of overlapping region to individual area of a structure was calculated and reported as a percent. One-way ANOVA with Dunnett's multiple comparisons test was performed for each colocalization group, and multiplicity adjusted p-values are shown. n = 40 shNT cells, 39 shG55 #06 cells, and 40 shG55 #63 cells, pooled from two experiments. Error bars: mean ± 95% confidence interval.

Biology Open • Supplementary information

Table S1. GRASP55 proximity-dependent biotinylation Proximity-dependent biotinylation was utilized to identify a GRASP55 proximal interactome. For each identity, the protein name, gene name, and primary corresponding UniProt ID are shown, along with the log_2-transformed intensity ratio of $GRASP55^{WT}$-BirA*-HA (WT) over $GRASP55^{G2A}$- BirA*-HA (G2A) samples and p-value (calculated by Welch's two-tailed unpaired t-test).

Protein name	Gene Name	UniProt ID	Log_2 (WT/G2A)	p-value
Thyroid receptor-interacting protein 11	TRIP11	Q15643	7.19194	0.00433
Zinc transporter 5	SLC30A5	Q8TAD4	5.55905	0.00817
Transmembrane emp24 domain-containing protein 7	TMED7	Q9Y3B3	4.35477	0.01864
Transmembrane emp24 domain-containing protein 9	TMED9	Q9BVK6	4.16124	0.02173
Zinc transporter 6	SLC30A6	Q6NXT4	4.12286	0.00337
Golgin subfamily A member 5	GOLGA5	Q8TBA6	3.90961	0.00500
Protein transport protein Sec24B	SEC24B	O95487	3.53728	0.02284
Syntaxin-5	STX5	Q13190	3.40771	0.01695
Transmembrane emp24 domain-containing protein 3	TMED3	Q9Y3Q3	3.18841	0.00043
Golgi resident protein GCP60	ACBD3	Q9H3P7	2.99792	0.00341
SEC23-interacting protein	SEC23IP	Q9Y6Y8	2.90721	0.00443
General vesicular transport factor p115	USO1	O60763	2.90585	0.02420
Zinc finger protein-like 1	ZFPL1	O95159	2.83137	0.00075
Transmembrane emp24 domain-containing protein 2	TMED2	Q15363	2.76387	0.01200
Transmembrane emp24 domain-containing protein 4	TMED4	Q7Z7H5	2.50645	0.00069
Protein transport protein Sec23B	SEC23B	Q15437	2.45113	0.00019
Transmembrane emp24 domain-containing protein 10	TMED10	P49755	2.35469	0.01919
Golgi pH regulator B	GPR89B	P0CG08	2.33330	0.00762
Histone H2A type 1-C	HIST1H2AC	Q93077	2.24515	0.00185
Protein transport protein Sec24A	SEC24A	O95486	2.07547	0.00314
Pyridoxal-dependent decarboxylase domain-containing protein 1	PDXDC1	Q6P996	2.06787	0.00019
Golgi SNAP receptor complex member 2	GOSR2	O14653	2.05011	0.01285
D-dopachrome decarboxylase	DDT	P30046	1.95804	0.03110
WW domain-containing oxidoreductase	WWOX	Q9NZC7	1.92233	0.00295
Protein transport protein Sec23A	SEC23A	Q15436	1.89144	0.00426
Protein YIF1A	YIF1A	O95070	1.70701	0.03173

Biology Open • Supplementary information

**Protein name**	**Gene Name**	**UniProt ID**	**Log$_2$ (WT/G2A)**	**p-value**
Membrane-associated progesterone receptor component 2	PGRMC2	O15173	1.68878	0.00386
Synaptobrevin homolog YKT6	YKT6	O15498	1.66690	0.00071
V-type proton ATPase subunit S1	ATP6AP1	Q15904	1.39232	0.01435
Acetyl-CoA acetyltransferase, cytosolic	ACAT2	Q9BWD1	1.30291	0.02083
Metalloreductase STEAP3	STEAP3	Q658P3	1.25844	0.02656
Coatomer subunit gamma-2	COPG2	Q9UBF2	1.15837	0.01516
WD40 repeat-containing protein SMU1	SMU1	Q2TAY7	1.15667	0.00207
NADH dehydrogenase [ubiquinone] flavoprotein 2, mitochondrial	NDUFV2	P19404	1.12821	0.04721
DDRGK domain-containing protein 1	DDRGK1	Q96HY6	1.08897	0.01576
Lamina-associated polypeptide 2, isoforms beta/gamma; Thymopoietin; Thymopentin	TMPO	P42167	1.07083	0.01372
Calumenin	CALU	O43852	1.04131	0.02810
Rhomboid domain-containing protein 2	RHBDD2	Q6NTF9	1.00934	0.04286

Table S2. Autophagy-related proteins detected by GRASP55 BioID

Selected autophagy-related proteins that were biotinylated by GRASP55 BioID constructs are shown. For each identity, the protein name, gene name, and primary corresponding UniProt ID are shown, along with the log_2-transformed intensity ratio of $GRASP55^{WT}$-BirA*-HA (WT) over $GRASP55^{G2A}$- BirA*-HA (G2A) samples and p-value (calculated by Welch's two-tailed unpaired t-test).

**Protein name**	**Gene Name**	**UniProt ID**	**Log_2 (WT/G2A)**	**p-value**
Sequestosome-1	SQSTM1	Q13501	0.15123	0.60510
Autophagy-related protein 9A	ATG9A	Q7Z3C6	0.05105	0.80999
Autophagy-related protein 2 homolog B	ATG2B	Q96BY7	-0.61061	0.04154
Ubiquitin-like-conjugating enzyme ATG3	ATG3	Q9NT62	-0.74869	0.10453
WD repeat domain phosphoinositide-interacting protein 2	WIPI2	Q9Y4P8	-0.86149	0.13794

Biology Open • Supplementary information
